# The Role of Genotypes That Modify the Toxicity of Chemical Mutagens in the Risk for Myeloproliferative Neoplasms

**DOI:** 10.3390/ijerph120302465

**Published:** 2015-02-24

**Authors:** Carol Ann Gross-Davis, Karyn Heavner, Arthur L. Frank, Craig Newschaffer, Judith Klotz, Regina M. Santella, Igor Burstyn

**Affiliations:** 1 Environmental Protection Agency, Region 3, 1650 Arch Street, Philadelphia, PA 19103, USA; 2 Department of Environmental and Occupational Health, Drexel University, Philadelphia, PA 19104, USA; E-Mails: karynkh@aol.com (K.H.); alf26@drexel.edu (A.L.F.); judith.klotz@comcast.net (J.K.); igor.burstyn@drexel.edu (I.B.); 3 A.J. Drexel Autism Institute, Drexel University, Philadelphia, PA 19104, USA; E-Mail: cnewscha@drexel.edu; 4 Department of Environmental Health Services, Mailman School of Public Health, Columbia University, New York, NY 10032, USA; E-Mail: rps1@cumc.columbia.edu

**Keywords:** case-control study, candidate gene, polycythemia vera (PV), essential thrombocythemia (ET), primary myelofibrosis (PMF), Mendelian randomization

## Abstract

*Background*: The etiology of myeloproliferative neoplasms (MPN) (polycythemia vera; essential thrombocythemia; primary myelofibrosis) is unknown, however they are associated with a somatic mutation—*JAK2* V617F—suggesting a potential role for environmental mutagens. *Methods*: We conducted a population-based case-control study in three rural Pennsylvania counties of persons born 1921–1968 and residing in the area between 2000–2008. Twenty seven MPN cases and 292 controls were recruited through random digit dialing. Subjects were genotyped and odds ratios estimated for a select set of polymorphisms in environmentally sensitive genes that might implicate specific environmental mutagens if found to be associated with a disease. *Results*: The presence of *NAT2* slow acetylator genotype, and *CYP1A2*, *GSTA1*, and *GSTM3* variants were associated with an average 3–5 fold increased risk. *Conclusions*: Exposures, such as to aromatic compounds, whose toxicity is modified by genotypes associated with outcome in our analysis may play a role in the environmental etiology of MPNs.

## 1. Introduction

Myeloproliferative neoplasms (MPNs), including polycythemia vera (PV), essential thrombocythemia (ET), and primary myelofibrosis (PMF), are rare cancers characterized by an overproduction of red blood cells and platelets [1]. Mandatory reporting on MPNs in the US began in 2001. The National Cancer Institute (NCI) estimated the incidence of PV, ET and PMF at 2.8, 1.5, and 0.4 cases per 100,000 person-years for 2001–2004 [2]. In 2008 the World Health Organization (WHO) revised the diagnostic criterion to include a diagnostic algorithm with genetic information (*JAK2* V617F) along with clinical information [3]. There are no known causes of MPNs although there have been a number of speculations about an environmental etiology [4].

The presence of the acquired *JAK2* V617F somatic point mutation—a single-base substitution resulting in a change from valine to phenylalanine at position 617 in the protein on chromosome 9p [5]—is suspected to be pathognomonic of PV. It is present in nearly all PV patients (>95%), about half of those with ET or PMF, and less frequently in other hematologic diseases but not in any other cancers [6,7,8]. JAK2 is a protein that acts as an on-and-off switch regulating bone marrow activity [9]. In the presence of the mutation, a tyrosine kinase complex in bone marrow, normally responsible for regulating blood cell production, is activated to increase blood cell production [6]. The causes of the *JAK2* V617F mutation are unknown [7].

There is strong evidence supporting familial risk for MPNs [4,10]. The role of inherited genetics has been suspected to influence MPN phenotype and susceptibility, including potential germ-line mutations yet to be identified [11]. Although functional genes that may modify the biological dose of a chemical mutagen have been studied for a wide range of cancers, no studies have investigated associations of these genotypes with MPNs [12]. Because of the role of the somatic *JAK2* V617F mutation in the etiology of MPNs, genes that modify susceptibility to mutagenic chemicals are of particular interest.

If we assume that there are genes that are not sufficient independent causes of MPNs but act exclusively to increase susceptibility to prevalent environmental mutagens, we can evaluate the main effect associations of these genes with MPNs to efficiently explore the potential role of the exposures whose effect they modify (*i.e.*, under these assumptions genotype associations infer existence of the gene-environment interactions) [13,14,15]. This approach is related to the Mendelian randomization that allows one to infer causation without directly measuring the exposure of interest by making the defensible assumption, in the given case, that genes are allocated independent of exposure and confounders [13]. The main objective of such analyses is not so much to quantify the extent of the gene-environment interaction but to use evidence of such interactions (assessed via genotype) to implicate specific exposures in the etiology of a disease.

The aim of the current analysis is thus to use this approach to identify potential classes of environmental exposures implicated in MPN etiology.

## 2. Methods

The study protocol was approved by the Drexel University and Geisinger Health Systems Institutional Review Boards. Participants received a $25 gift card for completing the phone survey phase and an additional $25 gift card if they gave a blood sample which was used in genotyping.

### 2.1. Selection of Cases and Controls

A case-control study was conducted in a tri-county area in Northeast Pennsylvania (Carbon, Luzerne, and Schuylkill counties) [16]. Cases and controls were eligible if they were born between 1921 and 1968 (*i.e*., 42–89 years old at the time of the study), resided in the tri-county area between 2000 and 2008 and completed a telephone survey. Cases were identified from the Pennsylvania Cancer Registry (PCR) using the International Classification of Diseases codes (ICD-O) for MPNs (codes for PV, M-9962/3; ETM, 9961/3; PMF, M-9931/3) as well as a PV cluster investigation in 2009 that included cases diagnosed between 2001 and 2006 in the three county area [17,18]. We included cases diagnosed up to 31 December 2010. We attempted to contact eligible cases that had relocated from the tri-county area. Controls were selected using a random digit dialing sample, stratified by age, sex and county to obtain distributions that reflected those of the source counties. Controls had to be free of MPNs during the time period that cases were ascertained. Among persons who consented to an interview, 57% of the cases and 62% of controls also provided DNA for the current analysis. Medical records of all cases not previously confirmed by the ATSDR cluster investigation [18] were evaluated by one of two expert panels (one created by the Pennsylvania Department of Health and one used in case ascertainment for a related study done by the University of Pittsburgh) [19]. Both panels used a similar methodology, and a two-thirds majority decision rule for case determination. Of the 31 cases genotyped and identified through the PCR and aforementioned ATSDR cluster investigation, 27 were confirmed as consistent with clinical records. These 27 cases of MPNs and 292 controls were eligible for the current analysis.

### 2.2. Selection of Environmentally Sensitive Genes

The National Institute of Environmental Health Sciences’ Environmental Genome Project (NIEHS EGP) has enumerated 647 known environmentally sensitive genes (ESG). We considered only genes identified by the NIEHS EGP that contained non-synonymous single nucleotide polymorphisms (nsSNPs), with a >5% minor allele frequency (MAF). Thus, 114 metabolism genes were given initial consideration, predominantly, the Phase I and Phase II detoxifying enzyme classes. The functional gene groups of interest regulate enzymes of two categories: metabolism or detoxification. Although other functional categories may be associated with the effect of chemicals of interest, they are out of the scope of this research. To facilitate the nsSNP selection, we used the Genome Variation Server (GVS) to obtain SNP information for nearly all EGP genes [20]. Only 82 of the 114 genes had coding SNP data in the GVS database. We reviewed the remaining 82 genes with nsSNP data for relevance to chemicals acting through a mutagenic pathway and the ability to be tested using the Illumina Bead Express platform. We ultimately included 21 genes using this platform for this project, in addition to the three genes without coding data, as shown in Table 1. In general, the variants included in this analysis modify enzyme function to magnify the mutagenic effect of the xenobiotic substrate [21] and include variants of the following genes: *AHR*, *ARNT*, *CYP1A1*, *CYP1A2*, *CYP1B1*, *CYP2B6*, *CYP2C9*, *CYP2E1*, *CYP3A5*, *CYP4B1*, *EPHX1*, *GSTA1*, *GSTP1*, *GSTM1*, *GSTM3*, *GSTT1*, *GSTZ1*, *MPO*, *NAT2*, *NQO1*, *OGG1*, *TP53* and *XRCC1* (Table 1) [20,22]. A tag SNP rs1495741 was included for *NAT2* and was used to infer NAT2 slow phenotype [23].

#### Genotyping

DNA was extracted from white blood cells by a standard salting-out protocol [24]. DNA quality was assessed by absorption at 260 and 280 nm. Samples were aliquoted into 96 well plates for analysis. Genotyping for all selected SNPs, except rs1048943 and rs4646903, was carried out using the Illumina Bead Express platform that employs VeraCode technology (Illumina, San Diego, CA, USA). Rs1048943 and rs4646903 were genotyped by TaqMan™ assays (LifeTechnologies/Applied Biosystems, Carlsbad, CA, USA) in a 384 well plate format using an Applied Biosystems 7900 PCR system. About 7% of samples were run in duplicate for both SNP genotyping assays. Deletions in *GSTM1* and *GSTT1* were determined using TaqMan Copy Number Assays™ with RNase P as the control gene. Samples were run in triplicate and CopyCaller™ Software was used for determination of the copy number. The bead express for the genotyping was run by Dr. Robin J. Leach, Co-Director of the Genomics Resource Core of the University of Texas, Health Services Center, School of Medicine, San Antonio. The *JAK2* V617F testing was completed by Dr. Mingjiang Xu at Mt. Sinai School of Medicine.

### 2.3. Statistical Analysis

Descriptive analysis was conducted on the characteristics of the study population. Logistic regression estimated adjusted odds ratios (OR) and associated 95% confidence intervals (CI). All ORs were adjusted for the design variables used to stratify the population for selection of controls (sex, age, county). We used the highest frequency of the homozygous genotype as the reference unless the literature indicated a different referent group (see Table A1). We conducted analysis of genotypes in the control population to evaluate Hardy-Weinberg equilibrium in the genetic variants. We applied “gene-only analysis” [13] to estimate the main effect of the gene of interest as a signal for gene-environment interaction. Each genotype was tested one at a time. We also restricted analysis to only confirmed PV cases as well as only *JAK2* V617F as additional case categories. In addition, an analysis that considered the total number of deleterious SNPs was also performed. Statistical analyses were conducted in SAS v. 9.2 (SAS Institute, Cary, NC, USA).

**Table 1:** Genes associated with a mutagenic chemical and minor allele frequency in the general population [19].

Genes	Function and Variant Effects (GVS)	Minor Allele Frequency (%)	Chemical Exposure
*AHR*	This gene encodes a ligand-activated transcription factor involved in the regulation of biological responses to planar aromatic hydrocarbons.	10	2,3,7,8-TCDD, benzo[a]pyrene 2,4'-DDT, Benzene
*ARNT*	The aryl hydrocarbon (Ah) receptor in xenobiotic Metabolism.	40	Dioxin and polycyclic aromatic hydrocarbons
*CYP1A1*	CYP1A1 is also known as AHH (aryl hydrocarbon hydroxylase). It is involved in the metabolic activation of aromatic hydrocarbons (PAH) some of which are found in cigarette smoke.	10	2,3,7,8-TCDD, sodium arsenite (NaAsO2), 2,4'-DDT, Aroclor-1260(weak), benzo[a]pyrene, benzo[k]fluoranthene, cadmium chloride
*CYP1A2*	The protein encoded by this gene localizes to the endoplasmic reticulum and its expression is induced by some PAHs, some of which are found in cigarette smoke. The enzyme’s endogenous substrate is unknown; however, it is able to metabolize some PAHs to carcinogenic intermediates. Other xenobiotic substrates for this enzyme include caffeine, aflatoxin B1, and acetaminophen.	24	2,3,7,8-TCDD, benzo[a]pyrene
*CYP1B1*	Metabolizes pro-carcinogens such as PAHs and 17beta-estradiol.	44	2,3,7,8-TCDD, benzo[a]pyrene
*CYP2B6*	This enzyme is known to metabolize some xenobiotics, such as the anti-cancer drugs cyclophosphamide and ifosphamide.	21	2,4'-DDT
*CYP2E1*	Inactivates a number of drugs and xenobiotics and also bioactivates many xenobiotic substrates including ethanol to their hepatotoxic or carcinogenic forms.	6	Benzene, methylene chloride, Styrene, 1,1,1-trichloroethane, Trichloroethylene
*CYP3A5*	In liver microsomes, this enzyme is involved in a NADPH-dependent transport pathway. It oxidizes a variety of structurally related compounds such as xenobiotics, fatty acids and steroids.	31	Drug metabolism and synthesis of cholesterol, steroids and other lipids
*CYP4B1*	Involved in metabolism of many xenobiotics including 2-aminofluorene, benzidine and 2-naphthylamine	29	Drug metabolism and synthesis of cholesterol, steroids and other lipids
*EPHX1*	Reduces enzyme function through metabolism of PAHs, and epoxide detoxification of 1,3-butadiene	33	PAH, 1,3-butadiene
*GSTM1*	This GST family member is a polymorphic gene encoding proteins that are active in xenobiotic metabolism including carcinogens, environmental toxins and therapeutic drugs	49	Styrene, benzene
*GSTA1*	This GST family member is a polymorphic gene encoding active, variant proteins that are thought to function in xenobiotic metabolism and in susceptibility to cancer	14	Hetero-Cyclic Amine (HCA)
*GSTM3*	This GST family member is a polymorphic gene encoding proteins that are active in xenobiotic metabolism and in susceptibility to cancer	32	PAH and HCA
*GSTP1*	This GST family member is a polymorphic gene encoding active, functionally different GSTP1 variant proteins that are thought to function in xenobiotic metabolism and play a role in vulnerability to cancer	32	Benzene
*GSTT1*	This GST family member is a polymorphic gene with a role suspected in human carcinogenesis	20	Benzo[a]pyrene, methylene chloride, Styrene, Benzene
*GSTZ1*	This GST family member is a polymorphic gene that encodes multifunctional enzymes involved in the detoxification of some carcinogens, mutagens, and several therapeutic drugs	5	Phenylalanine and tyrosine.
*MPO*	Xenobiotic metabolism and generates reactive oxygen species and can decrease normal enzyme function	11	Benzene, BaP, HCA
*NAT2*	Polymorphisms in this gene are responsible for the *N*-acetylation polymorphism in which human populations segregate into rapid, intermediate, and slow acetylator phenotypes. Polymorphisms in this gene are also associated with higher incidences of cancer and drug toxicity.	37	Benzo[a]pyrene, arylamine and hydrazine metabolism
*NQO1*	Mutations in this gene have been associated with tardive dyskinesia (TD), an increased risk of hematotoxicity after exposure to benzene, and susceptibility to various forms of cancer.	22	2,3,7,8-TCDD, benzo[a]pyrene
*OGG1*	Can result in decreased base excision DNA repair.	22	Aromatic hydrocarbons, most are repaired by NER not BER, this does repair 8-oxoG
*TP53*	Encodes a tumor suppressor protein and has an effect on cell-cycle regulation including DNA repair and changes in metabolism.	39	All genotoxic compounds
*XRCC1*	Involved in base excision repair and is linked to OGG1 gene.	12	Aromatic hydrocarbons

## 3. Results

The majority of MPN cases were confirmed to be primary PV (24/27). A greater proportion of cases were older (median age = 71 *vs.* 63 years.) and male (56% *vs.* 40%) compared to controls but otherwise were demographically similar (Table 2). The study population was entirely Caucasian with only two Latino controls. None of the cases and only six controls reported Jewish ancestry. All examined genes existed in Hardy Weinberg equilibrium (details not shown). The prevalence of *CYP1A2*, *GSTA1*, *GSTM3*, and *NAT2* risk genotypes in controls were 7%, 18%, 9% and 57%, respectfully, which is in agreement with the reported frequency in the literature (Table 1).

**Table 2:** Demographic characteristics of cases and controls.

Variable	All MPN Cases *n* = 27 (%)	*JAK2* V617F Positive Cases *n* = 22 (%)	PV Cases *n* = 24 (%)	Controls *n* = 292 (%)
County				
Carbon	4 (15)	4 (18)	4 (17)	31 (11)
Luzerne	12 (44)	10 (45)	10 (42)	177 (61)
Schuylkill	11 (41)	8 (36)	10 (42)	84 (29)
Age				
42–64	7 (26)	6 (27)	5 (21)	167 (57)
65+	20 (74)	16 (73)	19 (79)	125 (43)
Sex (male)	15 (56)	12 (55)	13 (54)	118 (40)
Race/ethnicity				
Non-Hisp. White	27 (100)	22 (100)	24 (100)	290 (99)
Latino, Hispanic	0	0	0	2 (1)
Marital status				
Married	18 (67)	15 (68)	15 (63)	183 (63)
Widowed	7 (26)	5 (23)	7 (29)	47 (16)
Currently single	2 (7)	2 (9)	2 (8)	62 (21)
Education				
Less than high school	2 (7)	2 (9)	2 (8)	5 (2)
High school/GED	13 (48)	11 (50)	13 (54)	106 (36)
Some college	7 (26)	5 (23)	5 (21)	104 (36)
Bachelors degree	2 (7)	2 (9)	2 (8)	36 (12)
More than bachelors	3 (11)	2 (9)	2 (8)	41 (14)
Household income				
Less than $20,000	3 (12)	3 (15)	3 (14)	38 (14)
$20,000—$35,000	9 (36)	7 (35)	9 (41)	67 (24)
$35,000—$50,000	2 (8)	2 (10)	2 (9)	54 (20)
$50,000—$75,000	6 (24)	4 (20)	6 (27)	67 (24)
More than $75,000	5 (20)	4 (20)	2 (9)	48 (18)
Don’t know or refused to answer	2	2	2	18

Results for associations of MPNs with the environmentally sensitive genes are summarized in Table 3. The crude estimates were very similar to the effect estimates adjusted for the design variables. It must be noted that all estimates of ORs had wide confidence intervals and any interpretation of the magnitude of the effect and reliability of the hypothesis test should be approached with considerable caution. Having the most common homozygous *CYP1A1* rs4646903, *CYP1A2*, *EPHX1* rs2234922 and *Tp53* alleles increased the odds of MPNs by four- to five-fold, with the point estimates of the odds ratios of 5.1, 4.1, 5.0, and 5.4, respectively. The *CYP3A5* rs776746 AA genotype increased the risk of having an MPN on average 9-fold. The *GSTA1* rs3957356 AA genotype was associated with an increase in effect estimates for any MPNs, with an average OR of 1.9. The *GSTM3* rs7483 AA genotype was associated, on average, with elevated risk of any MPNs (OR = 3.9). The *GSTM1* null and *GSTZ1*rs7972 AA genotypes followed a similar trend of doubling the odds of having an MPN, e.g., OR of 2.4 and 2.8, respectively. We found that the *NAT2* slow acetylator AA genotype (rs1495741) was, on average, three times more common than wild-type genotype among cases, e.g., for any MPNs (OR = 3.1). We were not able to test for all seven of the other *NAT2* SNPs shown in the literature to determine slow acetylator phenotype, but we were able to test for four of them. We observed a 2-fold increase for *CYP2E1* across all case definitions. A similar increased risk was found for individuals with *NQO1* rs1800566 AG (OR = 1.9) and a 3-fold increase for *ARNT* rs12410394 AG or GG genotypes with an OR of 3.2.

**Table 3:** Association of myeloproliferative neoplasm with environmentally sensitive gene in case-control analysis: Odds ratios and (95% confidence intervals), adjusted for age, sex, and county **^1^**.

Gene ^2^	SNP	At Risk Coding Sequence Variant	All MPN Cases (*n* = 27)	*JAK2* V617F Cases (*n* = 22)	PV Cases (*n* = 24)
***AHR***	rs2066853	AG	1.0 (0.4, 2.9)	1.3 (0.4, 3.8)	1.2 (0.4, 3.5)
***ARNT***	rs12410394	GG	0.4 (0.2, 1.0)	0.5 (0.2, 1.3)	0.4 (0.1, 1.0)
		AA	0.2 (<0.1, 1.8)	--	0.2 (<0.1, 2.0)
	rs12410394	AA	0.3 (<0.1, 2.4)	--	0.3 (<0.1, 2.8)
	rs12410394	GG	0.5 (0.2, 1.2)	0.6 (0.2, 1.6)	0.4 (0.2, 1.2)
		AG or GG	3.2 (0.4, 25.6)	--	2.9 (0.4, 23.3)
***CYP1A1***	rs1048943	CT	2.1 (0.5, 8.1)	2.4 (0.6, 9.5)	1.4 (0.3, 7.0)
	rs4646903	CC	5.1 (0.5, 57.5)	--	6.8 (0.6, 80.3)
		CT	1.5 (0.6, 4.2)	1.6 (0.5, 4.6)	1.4 (0.5, 4.0)
***CYP1A2***	rs762551	AA	0.8 (0.3, 1.7)	0.8 (0.3, 1.9)	0.8 (0.3, 1.9)
		CC	4.1 (1.3, 13.2)	3.9 (1.1, 13.8)	3.7 (1.0, 13.3)
***CYP1B1***	rs1056836	CC	1.9 (0.7, 5.4)	1.5 (0.5, 4.4)	1.5 (0.5, 4.5)
		CG	0.7 (0.2, 1.8)	0.4 (0.1, 1.3)	0.6 (0.2, 1.6)
***CYP2B6***	rs3745274	AC	1.1 (0.5, 2.6)	1.2 (0.5, 3.0)	1.3 (0.5, 3.2)
***CYP2C9***	rs1057910	AC	1.4 (0.4, 4.5)	1.2 (0.3, 4.5)	1.6 (0.5, 5.3)
	rs1799853	AG	0.9 (0.3, 2.5)	0.9 (0.3, 2.6)	0.6 (0.2, 1.9)
***CYP2E1***	rs2031920	AG	2.0 (0.4, 9.8)	2.4 (0.5, 12.3)	2.3 (0.4, 11.7)
	rs2070673	AT	0.7 (0.2, 2.0)	0.7 (0.2, 2.1)	0.6 (0.2, 1.9)
	rs6413432	AT	1.5 (0.6, 4.2)	2.0 (0.7, 5.6)	1.8 (0.6, 5.0)
***CYP3A5***	rs776746	AA	9.1 (0.5, 162.6)	11.0 (0.6, 200.1)	10.7 (0.6, 192.5)
		AG	0.9 (0.3, 3.4)	0.7 (0.2, 3.4)	1.1 (0.3, 4.2)
***CYP4B1***	rs2297810	AG	1.3 (0.5, 3.0)	1.2 (0.5, 3.1)	1.5 (0.6, 3.7)
***EPHX1***	rs1051740	GG	2.3 (0.6, 8.4)	2.4 (0.6, 8.9)	2.3 (0.6, 8.8)
		AG	1.6 (0.6, 3.9)	1.0 (0.4, 2.8)	1.3 (0.5, 3.2)
	rs2234922	GG	5.0 (0.7, 34.1)	3.7 (0.3, 39.9)	6.4 (0.9, 46.7)
		AG	0.9 (0.3, 2.2)	0.9 (0.3, 2.4)	1.0 (0.4, 2.7)
***GSTA1***	rs3957356	AA	1.9 (0.8, 4.7)	2.7 (1.0, 7.0)	2.3 (0.9, 5.9)
***GSTM1 Null***			2.4 (1.0, 5.9)	2.2 (0.8, 6.0)	2.5 (0.9, 6.5)
***GSTM3***	rs7483	AA	3.9 (1.2, 12.3)	4.5 (1.2, 16.0)	3.3 (1.0, 11.2)
		AG	1.2 (0.5, 3.1)	1.6 (0.6, 4.5)	1.0 (0.4, 2.6)
	rs1332018	CC	0.8 (0.3, 2.2)	0.6 (0.2, 2.1)	1.0 (0.3, 3.0)
		AC	0.2 (0.1, 0.7)	0.3 (0.1, 0.7)	0.3 (0.1, 0.9)
	rs1799735	TG	0.5 (0.2, 1.4)	0.4 (0.1, 1.6)	0.6 (0.2, 1.7)
***GSTM3***	rs7483	AA	3.5 (1.2, 10.0)	3.5 (1.1, 10.7)	3.3 (1.1, 10.3)
***GSTP1***	rs1695	AG	0.8 (0.4, 1.9)	0.7 (0.3, 1.8)	0.7 (0.3, 1.8)
	rs1138272	AA	1.2 (0.4, 4.0)	1.1 (0.3, 4.0)	1.0 (0.3, 3.8)
		AG	0.4 (<0.1, 2.9)	--	0.4 (0.0, 3.2)
***GSTT1 Null***			0.8 (0.3, 2.5)	0.5 (0.1, 2.1)	0.6 (0.2, 2.4)
***GSTZ1***	rs7972	AA	2.8 (0.3, 28.7)	4.0 (0.4, 41.2)	3.4 (0.3, 37.3)
		AG	1.4 (0.5, 3.9)	1.8 (0.6, 5.3)	1.6 (0.5, 4.8)
	rs1046428	AG	0.4 (0.2, 1.1)	0.3 (0.1, 1.0)	0.4 (0.1, 1.1)
***NAT2***	rs1041983	AA	0.6 (0.1, 2.5)	0.7 (0.1, 3.1)	0.3 (<0.1, 2.2)
	rs1495741	AA	3.1 (1.1, 8.7)	4.6 (1.3, 16.1)	3.5 (1.1, 10.8)
	rs1799929	AA	2.4 (1.0, 5.7)	3.4 (1.3, 8.4)	2.4 (1.0, 6.1)
	rs1799930	AA	0.3 (<0.1, 2.6)	0.4 (<0.1, 3.1)	--
	rs1801280	GG	2.8 (1.2, 6.5)	4.0 (1.6, 9.9)	2.9 (1.2, 7.1)
***NQO1***	rs1131341	AG	1.7 (0.4, 6.6)	2.3 (0.6, 8.8)	1.2 (0.2, 5.6)
	rs1800566	AA	1.6 (0.2, 14.4)	--	2.0 (0.2, 17.9)
		AG	1.9 (0.8, 4.3)	1.8 (0.7, 4.4)	1.7 (0.7, 4.1)
***OGG1***	rs1052133	CG	1.0 (0.4, 2.4)	0.9 (0.4, 2.4)	1.0 (0.4, 2.6)
		GG	0.6 (0.1, 4.7)	0.7 (0.1, 5.6)	0.6 (0.1, 5.3)
***Tp53***	rs1042522	GG	5.4 (1.4, 20.8)	8.0 (2.0, 33.0)	6.8 (1.7, 28)
		CG	1.0 (0.4, 42.3)	1.3 (0.5, 3.4)	1.0 (0.4, 2.6)
***XRCC1***	rs25487	AA	0.9 (0.3, 2.6)	1.4 (0.4, 5.0)	1.1 (0.3, 3.8)
		AG	0.5 (0.2, 1.3)	0.8 (0.3, 2.1)	0.5 (0.2, 1.4)
	rs25489	GG	0.8 (<0.1)	0.8 (<0.1)	0.7 (<0.1)
	rs1799782	AA	18.2 (1.0, 339)	-	-
		AG	1.6 (0.4, 6.1)	1.3 (0.3, 6.2)	1.1 (0.2, 5.3)

^1^ Odds ratios could not be calculated because there were no cases/controls with those variants for missing results and specifically, MPO rs2333277 genotype CC and XRCCI, rs25489 AG genotypes had no cases with any case definition; ^2^ Susceptible genes to mutagenic chemicals and Single Nucleotide Polymorphisms (SNPs) with rs number.

All but one case (97%) harbored at least two of the evaluated SNPs that signal association of the outcomes with exposure to xenobiotics (*AHR, CYP1E2, GSTM1, Tp53, GSTT1, GSTM3, CYP1A2, NAT2*, or *GSTA1* 30/31) compared to 63% of controls (Table 4). None of the results, either for individual genotype or presence of two or more of these SNPs varied materially in analyses restricted to only PV cases or to cases with confirmed *JAK2* somatic mutations.

**Table 4:** Total number deleterious genes, number and frequency for cases and controls.

Number of Deleterious Genes ^1^	Number and Frequency Deleterious Genes ^1^		Frequency Deleterious Genes ^1^	
	**Cases**	**Controls**	**Cases (%)**	**Controls (%)**
0	0	11	0	3.70
1	1	99	3.23	33.30
At least 2	30	182	96.78	63.00

**^1^** AHR, CY1A2, CY1E2, GSTA1, GSTM1, GSTM3, GSTT1, NAT2, TP53.

## 4. Discussion

After studying the main effects of 24 environmentally sensitive genes, we found that variants in *NAT2*, *CYP1A2*, *GSTA1*, and *GSTM3* were statistically significantly associated with MPN risk with ORs between 1.5 and 4. In addition, variants in *CYPA1, CYP2E1, CYP3A5, EPHX1, TP53, MPO, GSTZ1, ARNT*, and *NQO1* were associated with MPNs in this study with ORs between 1.4 and 9. While these results do not confirm gene-environment interaction for any one specific chemical, the findings encourage further explanation of the interaction hypothesis with respect to biological pathways and chemical exposures implicated by these genes. These same genes appear to be associated with the presence of the *JAK2* V617F mutation that is pathogenomic of MPNs.

To detect the potential for existence of gene-environment interactions, the main effects of genes were used in this analysis. We did not have measures of the exposures of interest available and therefore could not estimate interactions or stratified effects directly. If we assume that genes alone do not cause MPNs, but can only act by modifying the toxicity of an environmental exposure, then testing the main effect of the genes is an efficient way to generate evidence supporting qualitative gene-environment interaction [13,14]. Based on this reasoning, we are essentially assuming the existence of gene-environment interaction without an independent gene effect. A main gene effect without exposure is unlikely, based on the knowledge about the pathway for these diseases [6,7]. Therefore we have no reason to believe that MPNs are caused exclusively by the genotypes under investigation.

In our study sample, we did not find any associations with smoking or occupational exposure to polycyclic aromatic hydrocarbons with risk of developing an MPN [16]. However, perhaps paradoxically, our findings suggest that specific genotypes that modify the toxicity of these exposures may play a role in MPNs. The lack of an association of smoking with MPNs is consistent with the limited prior literature [4]. However, the existing literature on occupational and chemical exposures and MPN is neither specific, nor consistent [25,26,27,28,29].

Moore *et al.*, reported an association of bladder cancer with *NAT2* slow acetylator genotype and smoking intensity [30]. Since aromatic amines are detoxified by NAT2, interaction is biologically plausible. Thus, if carcinogens in tobacco smoke where implicated in MPNs, as they are in bladder cancer, then we should have detected the main effect of NAT2 in our study. However, absence of an effect from smoking *per se* suggests that compounds not important to the toxicity of tobacco smoke but affected by NAT2 should be scrutinized.

Functional SNPs associated with benzene exposure were also explored in this analysis: *CYP2E1, GSTM1, AHR*, and *GSTT1* variants modify the biological dose of benzene. In addition, Tp53 has been reported to be involved with benzene hematopoietic stem-cell toxicity in mice [31]. Effect estimates for *AHR* and *GSTT1* were essentially null but not for GSTM1 and Tp53. Work by Quiroga, Kaplan *et al.*, and Mele *et al.*, implicated benzene or petroleum products as risk factors [25,32,33]. Among analyses with the best available estimates of exposure to benzene, a pooled analysis of 29 cases of myelodysplastic syndrome (MDS), showed a monotonic-dose response relationship with cumulative exposure to benzene (odds ratio (OR) = 4.33 95% CI: 1.31, 14.3) but not for other subtypes including 30 myeloproliferative disease (MPD) cases (OR = 1.79, 95% CI: 0.68, 4.74) [29]. Work of Schnatter *et al.* implies that diagnostic heterogeneity may be complicating attribution to specific MPNs to environmental exposures [29]. Overall, our results are consistent with the supporting prior body of literature that suggests that benzene may be implicated in some MPNs, although clearly the evidence is far from being consistent and conclusive.

Our study was limited by the small number of cases. MPNs are rare hematological malignancies with only a limited number of cases available for recruitment—even under cluster outbreak conditions. Our study is most informative about PV cases only. We included a total of 24 PV cases, which is in the range of other studies of MPN etiology with case groups ranging from 10 to 133 [32,33] (see also *n* = 53 [31] and *n* = 30 [29]). Another limitation could be an unbalance in age and sex distribution of cases and controls. We attempted to control this by including these factors in the logistic regression. But residual confounding cannot be ruled out.

Although MPNs are classified at malignant, they became a reportable disease in the US only recently, in 2001, and in Pennsylvania only hospitals were able to report until recently, so there is the possibility of under-reporting of these MPNs to the cancer registry. There were also changes in the diagnostic criteria by the WHO in 2001 and again in 2008 [34]. The 2008 diagnostic criteria included molecular as well as histological information for diagnosis, including the *JAK2* V617F mutation [34]. Our ability to test for this mutation directly helped minimize outcome misclassification. Furthermore, by considering only individuals with *JAK2* mutation to be cases in some of our analyses, we reduced the possibly of outcome misclassification if one were to assume that persons with the mutation experience exposures that already placed them on pathway towards developing clinical MPN.

The misclassification of genotypes was not a concern in this study because our call rate was high (>95%), and we only had one SNP (*UGT1A*) that consistently did not perform well [35,36]. Our study is vulnerable to false positive discovery due to “multiple comparisons” [37]. However, unlike GWAS studies, we started with 648 genes of interest and only examined genes that met our *a priori* “plausible candidate”. Our selective genotypes were functional SNPs which directly affect the enzymatic activity of the gene, influencing biological pathways that affect metabolic activation or detoxification processes of metabolism for mutagenic chemicals. We did not correct for elevated type II error associations in applying a correction factor in the analysis.

## 5. Conclusions

Our research made two main pivotal assumptions. First, we assumed that genotype and environmental exposures in this study population are independent. Second, we assumed that disease risk will not vary with genotype for subjects without environmental exposure. From this, we exploit the most generic form of Mendelian randomization that individuals receive a random allocation of alleles from their parents. Gustasfon and Burstyn presented this method and concluded that when both of these assumptions are met, using data on genotype and disease jointly, with only knowledge of the prevalence of exposure without individual level data on exposure, can be a practical approach to investigate gene-environment interaction [14]. Through this approach, a signal of increased risk will only result if an environmental exposure is operating through gene-environment interaction [27]. Our findings therefore suggest that aromatic compounds and heterocyclic amines play a role in MPNs. Future research on the exposures (other than smoking) affected by the *NAT2*, *GST*, and *CYP* genes may be fruitful avenues to better evaluate the environmental etiology of MPNs.

## Disclaimer

This study was funded by a Cooperative Agreement/Earmark (CDC Research Grant Number: 1R01EH000640-01). It must be noted that this article’s contents are solely the responsibility of the authors and does not relate to the views of ATSDR or CDC. The research presented in this paper has been conducted by an employee of U.S. Environmental Protection Agency (EPA), Region III and was funded through the Centers for Disease Control and Prevention (CDC). Its contents are solely the responsibility of the authors and do not necessarily represent the official views of the CDC, EPA or the U.S. government.

## Appendix

**Table A1:** The Distribution of Variants of Environmentally Sensitive Genes in the Study Sample; Reference Group Identified by an asterix (*).

Susceptible Genotype SNP and rs Number ^1^	All MPN Cases ^2^ *n* = 27 (%)	*JAK2* V617F Cases ^2^ *n* = 22 (%)	PV Cases ^2^ *n* = 24 (%)	Controls *n* = 292 (%)
***AHR*** rs2066853				
AG	5 (19)	5 (23)	5 (21)	59 (20)
AA	0	0	0	1 (0 )
GG *	22 (81)	17 (77)	19 (79)	230 (79)
Missing	0	0	0	2
***ARNT*** rs12410394				
AA	1 (4)	0	1 (4)	33 (11)
AG *	19 (70)	15 (68)	17 (71)	135 (47)
GG *	7 (26)	7 (32)	6 (25)	122 (42)
Missing	0	0	0	2
*CYP1A2* rs762551				
AA*	11 (41)	9 (41)	10 (42)	145 (50)
AC*	11 (41)	9 (41)	10 (42)	128 (44)
CC	5 (19)	4 (18)	4 (17)	19 (7)
***CYP1B1*** rs1056836				
CC	9 (33)	7 (32)	7 (29)	52 (18)
CG	9 (33)	6 (27)	8 (33)	148 (51)
GG *	9 (33)	9 (41)	9 (38)	91 (31)
Missing	0	0	0	1
***CYP2B6*** rs3745274				
AC	17 (63)	14 (64)	16 (67)	164 (56)
AA *	10 (37)	8 (36)	8 (33)	128 (44)
***CYP2C9*** rs1057910				
AC	4 (15)	3 (14)	4 (17)	32 (11)
AA *	23 (85)	19 (86)	20 (83)	257 (89)
Missing	0	0	0	3
rs1799853				
AG	6 (22)	5 (23)	4 (17)	64 (22)
GG *	21 (78)	17 (77)	20 (83)	225 (78)
Missing	0	0	0	3
***CYP2E1*** rs2031920				
AG	2 (7)	2 (9)	2 (8)	12 (4)
GG *	25 (93)	20 (91)	22 (92)	275 (96)
Missing	0	0	0	5
rs2070673				
AT	5 (19)	4 (18)	4 (17)	74 (26)
AA	0	0	0	9 (3)
TT *	22 (81)	18 (82)	20 (83)	206 (71)
Missing	0	0	0	3
rs6413432				
AT	6 (22)	6 (27)	6 (25)	48 (17)
TT	0	0	0	2 (1)
AA *	21 (78)	16 (73)	18 (75)	240 (83)
Missing	0	0	0	2
***CYP3A4*** rs2740574				
AG	0	0	0	14 (5)
AA *	26 (100)	22 (100)	23 (100)	277 (95)
Missing	1	0	1	1
***CYP3A5*** rs776746				
AA	1 (4)	1 (5)	1 (4)	1 (0)
AG	3 (11)	2 (9)	3 (13)	38 (13)
GG *	23 (85)	19 (86)	20 (83)	252 (87)
Missing	0	0	0	1
***CYP4B1*** rs2297810				
AG	9 (33)	7 (32)	9 (38)	74 (26)
AA	0	0	0	3 (1)
GG *	18 (67)	15 (68)	15 (63)	212 (73)
Missing	0	0	0	3
***EHPX1*** rs1051740				
GG	4( 15)	4 (18)	4 (17)	27 (9)
AG	14 (52)	9 (41)	11 (46)	131 (45)
AA *	9 (33)	9 (41)	9 (38)	131 (45)
Missing	0	0	0	3
rs2234922				
GG	2 (7)	1 (5)	2 (8)	4 (1)
AG	7 (26)	6 (27)	7 (29)	85 (29)
AA *	18 (67)	15 (68)	15 (63)	200 (69)
Missing	0	0	0	3
***GSTA1*** rs3957356				
AA *	9 (33)	9 (41)	9 (38)	53 (18)
AG *	11 (41)	7 (32)	9 (38)	148 (51)
GG	7 26	6 (27	6 (25)	91 (31)
***GSTM1***				
Undetermined	0	0	0	1 (0)
0	20 (74)	16 (73)	18 (75)	155 (53)
1 *	7 (26)	6 (27)	6 (25)	116 (40)
2 *	0	0	0	20 (7)
***GSTM3*** rs7483				
AA	6 (22)	5 (23)	5 (21)	26 (9)
AG	11 (41)	10 (45)	9 (38)	124 (43)
GG *	10 (37)	7 (32)	10 (42)	137 (48)
Missing	0	0	0	5
rs1332018				
CC	7 (26)	5 (23)	7 (29)	45 (16)
AC	7 (26)	6 (27)	7 (29)	156 (54)
AA *	13 (48)	11 (50)	10 (42)	89 (31)
Missing	0	0	0	2
rs1799735				
TG	4 (15)	3 (14)	4 (17)	81 (28)
TT	0	0	0	8 (3)
GG *	23 (85)	19 (86)	20 (83)	200 (69)
Missing	0	0	0	3
***GSTP1*** rs1695				
				
AG	13 (48)	10 (45)	11 (46)	135 (47)
GG	0	0	0	33 (11)
AA *	14 (52)	12 (55)	13 (54)	122 (42)
Missing	0	0	0	2
rs1138272				
AA	4 (15)	3 (14)	3 (13	(35 12)
AG	1 (4)	0	1 (4 )	30 (10)
GG *	22 (81)	19 (86)	20 (83)	227 78
***GSTT1***				
3 *	0	0	0	1 (0)
Undetermined	0	0	0	1 (0)
0	4 (15)	2 (9)	3 (13)	46 (16)
1 *	22 (81)	20 (91)	21 (88)	197 (67)
2 *	1 (4)	0	0	47 (16)
***GSTZ1*** rs7972				
AA	1 (4)	1 (5)	1 (4)	6 (2)
AG	5 (19)	5 (23)	5 (21)	44 (15)
GG *	21 (78)	16 (73)	18 (75)	242 (83)
rs1046428				
AG	6 (23)	4 (19)	5 (22)	98 (34)
AA	0	0	0	9 (3)
GG *	20 (77)	17 (81)	18 (78)	184 (63)
Missing	1	1	1	1
***MPO*** rs2333277				
CC	0	0	0	1 (0.3)
AA *	27 (100)	22 (100)	24 (100)	289 (99)
Missing	0	0	0	2
***NAT2*** rs1041983				
GG *	13 (48)	12 (55)	12 (50)	140 (48)
AG *	12 (44)	8 (36)	11 (46)	116 (40)
AA	2 (7)	2 (9)	1 (4)	35 (12)
Missing	0	0	0	1
rs1495741				
GG	1 (4)	1 (5)	1 (4)	22 (8)
AG	4 (15)	2 (9)	3 (13)	104 (36)
AA *	22 (81)	19 (86)	20 (83)	164 (57)
Missing	0	0	0	2
rs1799929				
GG*	9 (33)	7 (32)	7 (29)	112 (38)
AG*	8 (30)	5 (23)	8 (33)	124 (42)
AA	10 (37)	10 (45)	9 (38)	56 (19)
rs1799930				
GG *	14 (52)	13 (59)	13 (54)	148 (51)
AG *	12 (44)	8 (36)	11 (46)	114 (39)
AA	1 (4)	1 (5)	0	29 (10)
Missing	0	0	0	1
rs1801279				
GG *	27 (100)	22 (100)	24 (100)	291 (100)
AG * or AA				
Missing	0	0	0	1
rs1801280				
GG *	11 (41)	11 (50)	10 (42)	58 (20)
AG *	9 (33)	6 (27)	9 (38)	127 (44)
AA	7 (26)	5 (23)	5 (21)	106 (36)
Missing	0	0	0	1
***NQO1***				
rs1131341				
AG	3 (11)	3 (14)	2 (8)	17 (6)
GG *	24 (89)	19 (86)	22 (92)	275 (94)
rs1800566				
AA	1 (4)	0	1 (4)	12 (4)
AG	12 (44)	10 (45)	10 (42)	87 (30)
GG *	14 (52)	12 (55)	13 (54)	193 (66)
***CYP1A1*** rs1048943				
CT	3 (12)	3 (14)	2 (9)	18 (6)
TT *	23 (88)	19 (86)	21 (91)	269 (94)
Missing	1	0	1	5
rs4646903				
CC	1 (4)	0	1 (4)	3 (1)
CT	6 (23)	5 (24)	5 (22)	46 (16)
TT *	19 (73)	16 (76)	17 (74)	239 (83)
Missing	1	1	1	4
***Tp53*** rs1042522				
GG	4 (15)	4 (18)	4 (17)	12 (4)
CG	10 (37)	9 (41)	9 (38)	117 (40)
CC *	13 (48)	9 (41)	11 (46)	160 (55)
***XRCC1*** rs25489				
AG	27 (100)	22 (100)	24 (100)	239 (84)
GG	0	0	0	7 (2)
AA *	0	0	0	37 (13)
Missing	0	0	0	9
rs1799782				
AA	3 (11)	2 (9)	2 (8)	1 (0.3)
AG	1 (4)	0	0	23 (7)
GG *	23 (85)	20 (91)	22 (92)	267 (91)
Missing	0	0	0	1
rs25487				
AA	4 (15)	4 (18)	4 (17)	42 (14)
AG	9 (33)	9 (41)	8 (33)	125 (43)
GG *	14 (52)	9 (41)	12 (50)	120 (41)
Missing	0	0	0	5

^1^. Susceptible genes to mutagenic chemicals and Single Nucleotide Polymorphisms (SNPs) with rs number; ^2^. All MPN cases include PV, ET and PMF, confirmed cases by expert panel, confirmed *JAK2* V617F mutation with PV, ET or PMF confirmed by expert panel, confirmed by expert panel PV cases only.

## References

[1] Campbell P.J., Green A.R. (2005). Management of polycythemia vera and essential thrombocythemia. Hematol. Am. Soc. Hematol. Educ. Progr..

[2] Kutti J., Ridell B. (2001). Epidemiology of the myeloproliferative disorders: Essential thrombocythaemia, polycythaemia vera and idiopathic myelofibrosis. Pathol. Biol..

[3] Vardiman J.W., Thiele J., Arber D.A., Brunning R.D., Borowitz M.J., Porwit A., Harris N.L., le Beau M.M., Hellström-Lindberg E., Tefferi A., Bloomfield C.D. (2009). The 2008 revision of the World Health Organization (WHO) classification of myeloid neoplasms and acute leukemia: rationale and important changes. Blood.

[4] Anderson L.A., Duncombe A.S., Hughes M., Mills M.E., Wilson J.C., McMullin M.F. (2012). Environmental, lifestyle, and familial/ethnic factors associated withmyeloproliferative neoplasms. Am. J. Hematol..

[5] Langabeer S., Ni Ainle F., Conneally E., Lawler M. (2007). Incidence and significance of the *JAK2* V617F mutation in patients with chronic myeloproliferative disorders. Ir. J. Med. Sci..

[6] Spivak J.L. (2010). Narrative review: Thrombocytosis, polycythemia vera, and *JAK2* mutations: The phenotypic mimicry of chronic myelofibrosis. Ann. Intern. Med..

[7] Kralovics R., Passamonti F., Buser A.S., Teo S., Tiedt R., Passweg J.R., Tichelli A., Cazzola M., Skoda R.C. (2005). A gain-of-function mutation of *JAK2* in myeloproliferative disorders. N. Engl. J. Med..

[8] Tefferi A., Thiele J., Orazi A., Kvasnicka H.M., Barbui T., Hanson C.A., Barosi G., Verstovsek S., Birgegard G., Mesa R. (2007). Proposals and rationale for revision of the World Health Organization diagnostic criteria for polycythemia vera, essential thrombocythemia, and primary myelofibrosis: Recommendations from an *ad hoc* international expert panel. Blood.

[9] Baxter E.J., Scott L.M., Campbell P.J., East C., Fourouclas N., Swanton S., Vassiliou G.S., Bench A.J., Boyd E.M., Curtin N. (2005). Acquired mutation of the tyrosine kinase *JAK2* in human myeloproliferative disorders. Lancet.

[10] Landgren O., Goldin L.R., Kristinsson S.Y., Helgadottir E.A., Samuelsson J., Bjiou G.S., Bench A.J., Boyd E.M. (2008). Increased risks of Curtolycythemia vera, essential thrombocythemia, and myelofibrosis among 24,577 first-degree relatives of 11,039 patients with myeloproliferative neoplasms in Sweden. Blood.

[11] Andrikovics H., Nahajevszky S., Koszarska M., Meggyesi N., Bors A., Halm G., Tordai A. (2010). *JAK2* 46/1 haplotype analysis in myeloproliferative neoplasms and acute myeloid leukemia. Leukemia.

[12] Thier R., Brüning T., Roos P.H., Rihs H.P., Golka K., Ko Y., Bolt H.M. (2003). Markers of genetic susceptibility in human environmental hygiene and toxicology: The role of selected CYP, NAT and GST genes. Int. J. Hyg. Environ. Health.

[13] Burstyn I., Kim H.-M., Yasui Y., Cherry N.M. (2009). The virtues of a deliberately mis-specified disease model in demonstrating a gene-environment interaction. Occup. Environ. Med..

[14] Gustafson P., Burstyn I. (2011). Bayesian inference of gene–environment interaction from incomplete data: What happens when information on environment is disjoint from data on gene and disease?. Stat. Med..

[15] Luo H., Burstyn I., Gustafson P. (2013). Investigations of gene-disease associations: Costs and benefits of environmental data. Epidemiology.

[16] Heavner K., Gross-Davis C.A., Frank A., Newschaffer C., Klotz J., Burstyn I. (2014). Working environment and myeloproliferative neoplasm: A population-based case-control study following a cluster investigation. Am. J. Ind. Med..

[17] Pennsylvania Cancer Registry (PCR) (2006). Appendix B: Title 28, Part III, Chapter 27 Communicable and Noncommunicable Diseases, Section 27.31.

[18] Seaman V., Jumaan A., Yanni E., Lewis B., Neyer J., Roda P., Hoffman R. (2009). Use of molecular testing to identify a cluster of patients with polycythemia vera in eastern Pennsylvania. Cancer Epidemiol. Biomark. Prev..

[19] Buchanich J., Mertz K. (2013). Updated and expanded study of polycythemia vera and other myeloproliferative neoplasms in the tri-county area. Final Report to Pennsylvania Department of Health.

[20] GVS:Genome Variation Server, GVS. http://gvs.gs.washington.edu/GVS141/.

[21] Dong L.M., Potter J.D., White E., Ulrich C.M., Cardon L.R., Peters U. (2008). Genetic susceptibility to cancer. JAMA.

[22] Hung R.J., Boffetta P., Brockmoller J., Butkiewicz D., Cascorbi I., Clapper M.L., Garte S., Haugen A., Hirvonen A., Anttila S. (2003). CYP1A1 and GSTM1 genetic polymorphisms and lung cancer risk in Caucasian non-smokers: A pooled analysis. Carcinogenesis.

[23] Garcia-Closas M., Rothman N., Figueroa J.D., Prokunina-Olsson L., Han S.S., Baris D., Jacobs E.J., Malats N., de Vivo I., Albanes D. (2013). Common genetic polymorphisms modify the effect of smoking on absolute risk of bladder cancer. Cancer Res..

[24] Miller S.A., Dykes D.D., Polesky H.F. (1988). A simple salting out procedure for extracting DNA from human nucleated cells. Nucleic Acids Res..

[25] Kaplan S.D. (1986). Update of a mortality study of workers in petroleum refineries. J. Occup. Med..

[26] Zoloth S.R., Michaels D.M., Villalbi J.R., Lacher M. (1986). Patterns of mortality among commercial pressmen. J. Natl. Cancer Inst..

[27] Johnson E.S., Zhou Y., Lillian Yau C., Prabhakar D., Ndetan H., Singh K., Preacely N. (2010). Mortality from malignant diseases—Update of the Baltimore union poultry cohort. Cancer Causes Control.

[28] Pasqualetti P., Casale R., Colantonio D., Collacciani A. (1991). Occupational risk for hematological malignancies. Am. J. Hematol..

[29] Schnatter R., Glass D.C., Tang G., Irons R.D., Rushton L. (2012). Myelodysplastic syndrome and benzene exposure among petroleum workers: An international pooled analysis. J. Natl. Cancer Inst..

[30] Moore L.E., Baris D.R., Figueroa J.D., Garcia-Closas M., Karagas M.R., Schwenn M.R., Johnson A.T., Lubin J.H., Hein D.W., Dagnall C.L. (2011). GSTM1 null and NAT2 slow acetylation genotypes, smoking intensity and bladder cancer risk: Results from the New England bladder cancer study and NAT2 meta-analysis. Carcinogenesis.

[31] Hirabayashi Y. (2005). p53-dependent gene profiling for reactive oxygen species after benzene inhalation: Special reference to genes associated with cell cycle regulation. Chem. Biol. Interact..

[32] Quiroga Micheo E., Calcagno E.J., Calabria S.I., Besuschio S.C., Magnasco J.H., Sackmann Muriel F., Maccione E., Barros C., Santoro A., de Soto Z.C. (1981). Retrospective epidemiological study of hemopoietic system neoplasms in Argentina. Medicina.

[33] Mele A., Visani G., Pulsoni A., Monarca B., Castelli G., Stazi M.A., Gentile G., Mandelli F. (1996). Risk factors for essential thrombocythemia: A case–control study. Cancer.

[34] Vakil E., Tefferi A. (2011). BCR-ABL1—Negative myeloproliferative neoplasms: A review of molecular biology, diagnosis, and treatment. Clin. Lymphoma Myeloma Leuk..

[35] Smith G.D., Palmer L., Burton P. (2011). An Introduction to Genetic Epidemiology.

[36] Deitz A.C., Rothman N., Rebbeck T.R., Hayes R.B., Chow W.H., Zheng W., Hein D.W., García-Closas M. (2004). Impact of misclassification in genotype-exposure interaction studies: Example of N-acetyltransferase 2 (NAT2), smoking, and bladder cancer. Cancer Epidemiol. Biomark. Prev..

[37] Hunter D.J. (2005). Gene–environment interactions in human diseases. Nat. Rev. Genet..

